# Impact of Brain Lesion Characteristics on Motor Function and Cortical Reorganization in Hemiplegic Cerebral Palsy

**DOI:** 10.3390/medicina61020205

**Published:** 2025-01-24

**Authors:** Katerina Gaberova, Iliyana Pacheva, Nikolay Sirakov, Elena Timova, Ivan Stefanov Ivanov

**Affiliations:** 1 Department of Pediatrics, Medical Faculty, Medical University of Plovdiv, 4000 Plovdiv, Bulgaria; iliyana.pacheva@mu-plovdiv.bg (I.P.); ivan.ivanov@mu-plovdiv.bg (I.S.I.); 2 Department of Imaging Diagnostics, Dental Allergology and Physiotherapy, Dental Faculty, Medical University of Plovdiv, 4000 Plovdiv, Bulgaria; nikolay.sirakov@mu-plovdiv.bg; 3 Research Institute at Medical University of Plovdiv, 4000 Plovdiv, Bulgaria; etimova@gmail.com

**Keywords:** cerebral palsy, lesion characteristics, motor reorganization

## Abstract

*Background and Objectives*: Hemiplegic or unilateral cerebral palsy (UCP) is primarily characterized by motor impairment, mainly affecting the upper limb. Research has centered on factors influencing the varying degrees of motor deficit in UCP, using neuroscience advancements for in vivo exploration of brain structure (morphometry) and cortical reorganization (functional magnetic resonance imaging (fMRI)). This study aims to evaluate functional activation in the motor cortex in UCP and to explore how lesion characteristics and timing affect neuroplasticity and motor function. *Materials and Methods*: Between 2017 and 2021, structural and functional MRIs were performed on 44 UCP patients (mean age 15.5 years, 24 males, 20 females), all with Manual Ability Classification System (MACS) levels I-III and Intelligence Quotient (IQ) ≥ 50. The lesion characteristics of size, type, and time of occurrence (ante-, peri-, or early postnatal) were analyzed. An association was sought between the characteristics of the lesion and the degree of motor deficit of the upper limb, as determined by the MACS level. fMRI assessed cortical activation during a finger-tapping task for the paretic hand and compared activation patterns based on lesion characteristics. *Results*: Six lesion types were identified, with arterial ischemic stroke being the most common and largest in volume. Lesion size strongly correlated with patients’ MACS levels, while lesion type and timing showed no association with the severity of motor impairment classified by MACS. Motor reorganization varied, with activation occurring ipsi-, contra-, or bilaterally to the affected hand, depending on lesion size and type. Smaller, subcortical lesions primarily showed ipsilesional activation, while larger, cortical lesions did not exhibit a specific group activation, possibly due to varying individual reorganization. No association was found between the lesion timing and the reorganization model. *Conclusions*: Motor functional reorganization in UCP is closely linked to lesion characteristics, with smaller, subcortical lesions favoring typical organization in the contralateral motor cortex. The timing of the lesion does not significantly affect cortical reorganization. Lesion size was a key determinant of motor function, whereas lesion type (e.g., ischemic stroke) and timing (early vs. late occurrence) were less critical for predicting functional outcome.

## 1. Introduction

Cerebral palsy (CP) is a group of permanent disorders of the development of movement and posture, causing activity limitations that are attributed to non-progressive lesions in the developing fetal or infant brain [1]. Hemiplegic or unilateral cerebral palsy (UCP) results from damage to any part of the corticospinal pathway during its early development, with the morphology of the lesion varying depending on the timing of its occurrence. Cerebral malformations are most common during the embryonic period, periventricular lesions typically occur between 24 and 34 weeks, and arterial ischemic stroke (AIS) are more likely around full-term birth [2,3]. Early postnatal lesions (traumatic brain injuries, infections, or other acquired conditions) have various morphology [3,4]. The diversity of lesions causing UCP raises the question of how lesion morphology impacts the patient’s functional outcomes later in life [5]. Studies on lesion types have shown that worse upper limb function is observed in cortico-subcortical infarcts, as well as in larger lesions and those involving the posterior limb of the internal capsule and the basal ganglia [6,7,8,9,10]. Regarding the timing of lesions, most authors suggest that earlier-occurring lesions are associated with a better prognosis. In Cioni’s proposed classification, anyway, the prognosis for both early malformative lesions and early acquired lesions is generally regarded as unfavorable [3]. While some evidence highlights the significance of these structural and biological factors, a widely noted observation is the absence of a clear direct relationship between lesions and specific hand function outcomes in individuals with UCP [11]. Some authors, however, question whether motor function can be predicted solely by the morphology of the lesions. They suggest that functional reorganization may play a key role in the future motor performance of patients with UCP [8,9,12,13].

Damage to elements of the corticospinal pathway during the early stages of brain development can disrupt its typical progression. Normally, during the prenatal period and into the early postnatal stage (up to 18 months corrected age), bilateral corticospinal projections exist, meaning each limb is innervated by both hemispheres. Through synaptic pruning, ipsilateral fibers gradually undergo involution, so that by the age of two, no more than 15% of ipsilateral projections remain. However, in the presence of a lesion, the activity of ipsilateral synapses exceeds that of the contralateral ones, leading to involution of the latter, which deviates from the typical developmental pattern [14]. Thus, the ipsilateral cortex may exert some motor control over the distal musculature. The potential for preserving ipsilateral projections also influences the occurrence of three patterns of cortical reorganization following unilateral damage: contralateral, ipsilateral, or bilateral [15,16]. Evidence suggests that preserving motor function in the ipsilesional areas (contralateral to the affected limb) typically results in greater motor functional capacity compared to activation in contralesional or bilateral regions [17]. For children with large lesions, ipsilesional reorganization may not be feasible [18]. In such cases, contralesional reorganization may be the only viable option, which is not maladaptive but rather essential for preserving residual function [9]. The relationship between the type of cortical-motor organization and lesion morphology has been the focus of only a limited number of studies. The results indicate that smaller lesions promote ipsilesional functional reorganization, whereas larger lesions are more likely to result in interhemispheric reorganization within the “healthy” contralesional hemisphere [17,19]. Lesion type is often analyzed in relation to timing, as specific types of damage are associated with different developmental periods [2,3]. Most studies identify a connection between lesion timing and neuroplastic patterns in the motor cortex, suggesting that earlier-acquired lesions have a greater potential for ipsilesional reorganization (within the lesioned hemisphere) [2,20]. Due to the limited number of studies and the diverse nature of brain lesions leading to UCP, the relationship between cortical-motor reorganization and lesion characteristics remains unclear.

In conclusion, upper limb function in UCP exhibits significant variability, which cannot be attributed to a single characteristic of the lesion. Numerous neurobiological factors, including cortical-reorganization patterns, are likely to contribute to determining the patient’s clinical function. This study aims to investigate brain neuroplasticity following unilateral brain injury (occurring ante-, peri-, or postnatally), assess the impact of various factors—lesion size, type, and timing of occurrence—on cortical reorganization, and determine how these morphological characteristics relate to motor performance in patients with UCP.

## 2. Materials and Methods

### 2.1. Participants

The study was conducted at the Pediatrics Department of University Hospital ‘St. George’ in Plovdiv and the Translational Neuroscience Complex of the Medical University of Plovdiv, from 1 March 2021, to 31 March 2023. The study focused on children and young adults diagnosed with Congenital Hemiplegia according to the International Classification of Diseases (ICD-10) criteria. In total, 46 patients with a mean age of 15 years and 7 months (min was 9 years and 2 months, max was 23 years and 10 months) participated in the study. The work has been carried out following the Code of Ethics of the World Medical Association (Declaration of Helsinki) for experiments involving human subjects. The project was approved by the Committee on Scientific Ethics at the Medical University of Plovdiv /No1/25.02.2021/. Informed consent was obtained from either the participants (if they were of legal age) or their parents.

The inclusion criteria were as follows: a diagnosis of Congenital Hemiplegia (ICD-10); functional assessment using Manual Ability Classification System (MACS) levels 1–3 and Intelligence Quotient (IQ) ≥ 50 (measured by the Wechsler IQ test); and a unilateral brain lesion confirmed by previous brain imaging (Computed Tomography or Magnetic Resonance Imaging—MRI). The limitations for MACS ≤ 3 and IQ ≥ 50 were implemented because of the nature of the study, which involves functional MRI and requires patient cooperation. Patients with more severe motor involvement (MACS IV and V) often experience upper limb contractures, which prevent them from performing the finger opposition task. Regarding IQ, it was crucial for the MRI task that the participants could understand, read, and follow the instructions on the screen. The exclusion criteria included as follows: refusal to participate by the patient or parents; inability to provide informed consent due to a lack of understanding of the study; contraindications for MRI (e.g., metal implants, pacemakers, dentures); claustrophobia or fear preventing MRI; and inability to understand or perform functional tasks in MRI due to severely impaired hand function, severe intellectual disability, or behavioral issues. This limitation of the group (not including individuals with severe intellectual disabilities) could introduce a bias that may lead to differences when compared with other studies.

### 2.2. Clinical Determinants

A detailed history was obtained from all participants and their parents to identify risk factors for unilateral lesions, as well as concomitant conditions and the patient’s daily motor performance. The history was gathered through interviews and reviewed medical documentation, covering the mother’s pregnancy, delivery, early postnatal period, and the patient’s neurodevelopment. The identified risk factors for CP were categorized based on their timing into early and late prenatal, intranatal, and postnatal factors.

The clinical examination was conducted by a pediatric neurologist and included somatic and detailed neurological assessments, to confirm the diagnosis of UCP, following the ICD-10 definition for diagnosis G80.2. The MACS scale was used to evaluate the patients’ motor function, enabling a standardized digital assessment of upper limb usage. Participants in this study had MACS scores ranging from I to III, without severe upper limb contractures, ensuring their ability to perform the motor paradigm involving repetitive thumb opposition against the other fingers. Psychometric measurements were administered by a certified psychologist using the Wechsler Scale, and the results were employed as an inclusion criterion.

### 2.3. Structural Magnetic Resonance Imaging

Magnetic resonance imaging studies were conducted at the Complex of Translational Neuroscience at the Medical University of Plovdiv. The research protocol included structural neuroimaging with the 3D T1 FSPGR series, using a slice thickness of 1 mm, a matrix of 256 × 256, and a flip angle of 12°. During the study, a radiologist reviewed the images to confirm the presence of a unilateral lesion and determine the type of lesion. Lesions were classified based on their predominant subcortical or cortical involvement and further categorized using the Magnetic Resonance Imaging Classification System (MRICS).

For statistical processing of structural data, T1 DICOM images were converted to 3D.nii format using the MriCron program (https://www.nitrc.org/projects/mricron, accessed on 1 September 2021). Data analyses were performed using SPM (Statistical Parametric Mapping) version 12, installed on the MATLAB R2015a Windows platform, version 8.5. The 3D.nii files for each patient were normalized by transforming them into standard MNI (Montreal Neurological Institute) space and then segmented to classify different tissue types. To enable group-level statistical analyses, all images were reoriented so that the structural lesion appeared in the left hemisphere. This transformation was performed using the SPM “Reorient images” function for patients with original right-sided lesions. To differentiate lesions for each patient, the free software lesion_gnb [21] was utilized, which applies the unified segmentation method to delineate chronic lesions in T1 images. This process generates a lesion mask, which was subsequently used for volume determination using the “Extract values of interest” function, part of the LST tool, version 3.0.0 [22]. The resulting numerical lesion size values (in milliliters) were then incorporated into additional statistical analyses. The automatic methods used can reduce the possibility of human error, manipulation of results, or interindividual variability, and also minimize the potential impact on the final statistical results.

### 2.4. Functional Magnetic Resonance Imaging (fMRI)

The functional series were conducted in the same session as the structural MRI studies. Before the procedure, participants were familiarized with the process and the finger-tapping paradigm through an animated PowerPoint presentation. After the pre-procedural instruction, they underwent an assessment to verify their comprehension. The finger-opposition task is a motor task used to assess the fine motor skills of the hand, specifically the ability to oppose the thumb to each of the other fingers in a repetitive manner. It is widely used in research to access activation in the motor areas owing to their simplicity of construction and execution, and its utility in assessing motor function. The task has also been used in UCP patients [23]. The technical specifications for the functional neuroimaging series were as follows: 2D EPI series, slice thickness of 3 mm, matrix 96 × 96, repetition time (TR) of 3 s, echo time (TE) of 30, and a flip angle of 90°. Each series lasted 5 min and 15 s, beginning with 5 dummy scans (15 s) to stabilize the magnetic field. The series was presented using an E-prime paradigm, designed in a standard block format with alternating 30 s active and inactive blocks. The visual stimulus was projected to the participant through glasses connected to the control panel. At the start of each active block, and for the following 30 s, a visual stimulus indicating either “Start left hand” or “Start right hand” (depending on the affected side) was displayed to the participant. During this time, participants began performing repetitive thumb opposition against the other fingers of the affected hand (finger tapping) at a consistent rate of 1 tap per second. When the visual stimulus changed to “Stop,” participants had to cease hand movement and remain still. During the fMRI task, participants were closely monitored by the team members in the control room to visually confirm that they were performing the task correctly.

The processing of data from functional studies followed several stages as follows:

1. Converting the images from DICOM to nifty format through the MRICron program.
2. Transforming the images with a left-sided structural lesion into mirror images, visualizing the lesion into the right hemisphere through the SPM function “Reorient images”.
3. Realignment—correction of differences in time series, caused by head movements in a reference image—the average image of all time series.
4. Coregistration—between high-resolution structural images and functional images.
5. Normalization—transforming co-registered images into a standard MNI (Montreal Neurological Institute) space.
6. Smoothing—with Gaussian kernel 6 × 6 × 6 mm to smooth out differences in functional anatomy and to improve the signal-to-noise ratio.

### 2.5. Statistical Analysis

After completing the pre-processing steps (as described), the first (intra-individual) level of statistical analysis was performed using SPM12. The statistical model was specified, including the interscan interval (TR), the start time of each block, and the block duration. Active and passive conditions were contrasted. Multiple regressors, including motion parameters (from the corresponding realignment file), were added to the model. Scans with excessive head movement (greater than 2 mm in the x, y, and z directions) were excluded from the analysis using the Art tool, a software for detecting artifacts in fMRI data. As a result, a statistical parametric map of the Blood Oxygen Level Dependent (BOLD) signals in individual voxels was generated. After assessing statistical significance, t-contrasts between active and passive blocks were calculated in both directions (active > passive and passive > active) to identify which voxels showed significantly more activity during each condition.

At the second (inter-individual, group) level of statistical analysis in SPM12, the contrast maps from the previous step for each participant were subjected to random effects analysis to explore intergroup differences in the BOLD signal. The following methods were used:

- One-sample t-test: To assess statistically significant differences in average brain activation within a group.
- Independent two-sample t-test: To compare brain activation between two groups.
- Correlation test: To examine the relationship between brain activation and quantitative factors (such as lesion size, IQ, lesion onset time, etc.)

For all analyses, t-contrasts from the first level (active > passive) were applied. A significance level of *p* < 0.05 was used, with corrections for multiple comparisons using FWE (family-wise error) and a minimum cluster size of 5 voxels.

## 3. Results

### 3.1. Sample Characteristics

Patients with right-sided hemiparesis were prevalent, with 30 out of 44 patients (66.18%) compared to 14 out of 44 patients (33.81%) with left-sided hemiparesis. The MACS classification for patients was as follows: 21/44 (47.7%) had MACS I, 6/44 (13.6%) had MACS II, and 17/44 (38.6%) had MACS III. The Wechsler test revealed an average IQ of 78 in the patient group (ranging from 50 to 103), with verbal intelligence averaging 79.5 (ranging from 50 to 105), and nonverbal intelligence averaging 76 (ranging from 50 to 105). A high proportion of patients experienced concomitant conditions. Among them, epilepsy was observed in 20 out of 44 patients (45.5%), with therapeutic remission (seizure-free for the past two years) achieved in 11 of the 20 cases (55%). Sensory deficits were noted in 15 out of 44 patients (32%), with stereognosis and graphesthesia being the most commonly affected (8 out of 15 cases). Minor dystonia affecting the paretic upper limb was identified in 2 out of 44 participants (4.5%). The average age for initiating physical therapy was 13 months, ranging from a minimum of 4 months to a maximum of 24 months, determined by the timing of diagnosis. The average frequency of physical therapy sessions was once a week, varying from daily to once a month. Table 1 summarizes the individual characteristics of the participants.

### 3.2. Lesion Characteristics

Lesions were classified based on the MRICS and further categorized into those involving both the cortex and subcortical white matter and those confined to subcortical structures only. The individual characteristics of the lesions are detailed in Table 1. AIS represented the largest proportion of all lesion types (36.4%) (chi-square = 24.091; *p* < 0.001). All B lesions (B1: periventricular leukomalacia (PVL), B2: sequelae of intraventricular hemorrhage (IVH) or periventricular hemorrhagic infarction (PVHI), and B3: combination of PVL and PVHI sequelae) were subcortical, as well as all C1 lesions (basal ganglia/thalamus infarctions), while C3 lesions (AIS in the middle cerebral artery), A1 lesions (disorders of cortical formation), and three out of four D lesions (miscellaneous) were cortical.

Analysis of the effect of lesion type on clinical performance (assessed using MACS level, mirror movements (MM), and hand-clapping speed) revealed only one significant association: lesser MM performance in patients with subcortical lesions (Pearson chi-square = 4.956; *p* < 0.05). The lesion type (whether determined as cortical or subcortical, or classified by MRICS) did not significantly influence hand motor performance, as assessed by the MACS level or hand-clapping speed, an additional measure of hand motor function. The results are provided in the Appendix A (Table A1 and Table A2).

**Figure 1:**
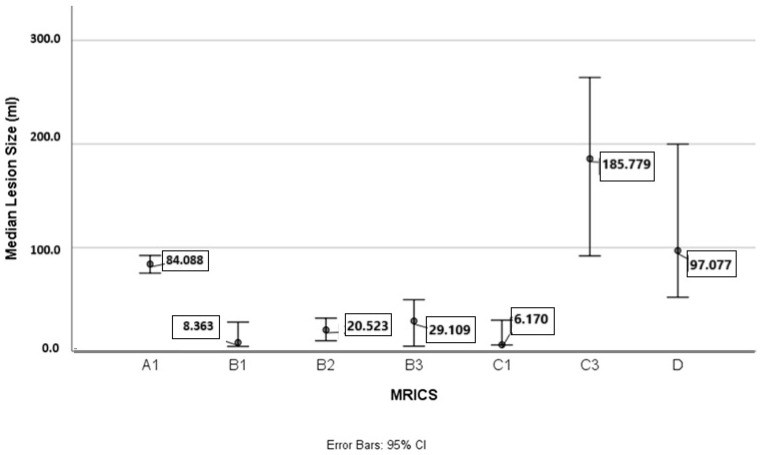
Median lesion size (in milliliters, ml, vertical axis) across different MRICS (Magnetic Resonance Imaging Classification System) categories (horizontal axis). A1: disorders of cortical formation; B1: periventricular leukomalacia; B2: sequelae of intraventricular hemorrhage or periventricular hemorrhagic infarction; B3: combination of periventricular leukomalacia and periventricular hemorrhagic infarction sequelae; C1: basal ganglia/thalamus infarctions; C3 lesions: arterial ischemic infarction in the middle cerebral artery; D: miscellaneous. Each point is accompanied by error bars representing the 95% confidence interval (CI) for the median lesion size in each category.

The average lesion size in the studied group was 48.8 mm^3^ (range: from 4.6 mm^3^ to 422.1 mm^3^). The average lesion size by MRICS type is presented in Figure 1, with individual results shown in Table 1. A significant difference in lesion size was found between groups (*p* < 0.001), with AIS being the largest lesions, while subcortical lesions (PVL, PVHI, and basal ganglia infarction) were the smallest.

A larger lesion was found to correlate positively with more severe motor deficit, evaluated as MACS level, as well as with the presence of MM. As lesion size increased, the severity of hand motor impairments worsened, accompanied by a higher frequency and intensity of pathological synkinesis. No correlation was found with the hand-clapping speed. Results are shown in Table 2.

Based on the MRICS imaging classification and risk factors from the patient’s history, the possible timing of the lesion occurrence was determined as follows: <24 weeks gestational age (GA)—brain maldevelopments (n = 3); 24–34 weeks GA—periventricular lesions (PVL and PVHI), and two patients with AIS and prematurity (n = 20); >34 weeks GA—AIS, including basal ganglia infarctions (n = 15); postnatal lesions (n = 6)—including postnatal ischemic strokes, encephalitis, and traumatic brain hemorrhages. A significant difference in lesion size was found between the four groups (Kruskal–Wallis H = 25.8; *p* < 0.001), with the largest lesions occurring in the perinatal group, followed by the postnatal group.

The chi-square analysis did not reveal any significant association between the timing of the brain lesion and any of the clinical parameters evaluated (MACS level, hand-clapping speed, or MM). This indicates that the period of lesion occurrence, categorized into the four defined timeframes, did not have a substantial impact on the motor performance of the participants.

### 3.3. fMRI Results

**Figure 2:**
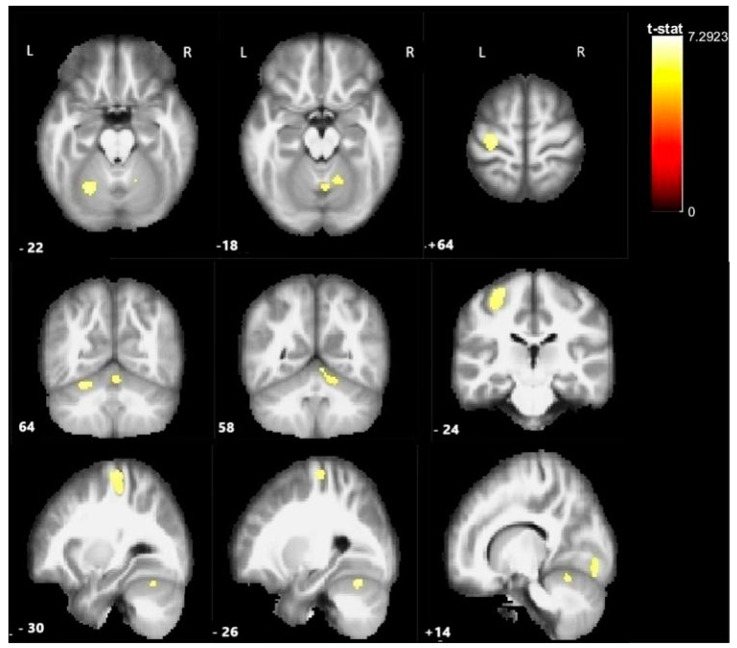
Statistical parametric maps of activation during motor paradigm with the paretic (unified right hand) in patients with UCP show significant activation in the left (ipsilesional) motor cortex and the cerebellum bilaterally. Statistical parametric maps of activation are overlaid into a standard MNI template. L: left (ipsilesional) hemisphere; R: right (contralesional hemisphere). BSPMview toolbox (http://www.bobspunt.com/software/bspmview/, accessed on 21 September 2021) was used for the results presentation, with a threshold set at *p* < 0.05 (FWE-corrected). The color bar represents T-values.

Overall group analysis revealed significant activation in five clusters: the left (ipsilesional) precentral gyrus, lobule I and lobule VI of the left cerebellum, lobule VI of the right cerebellum, and the cerebellar vermis. The results are presented in Table 3 and Figure 2.

**Figure 3:**
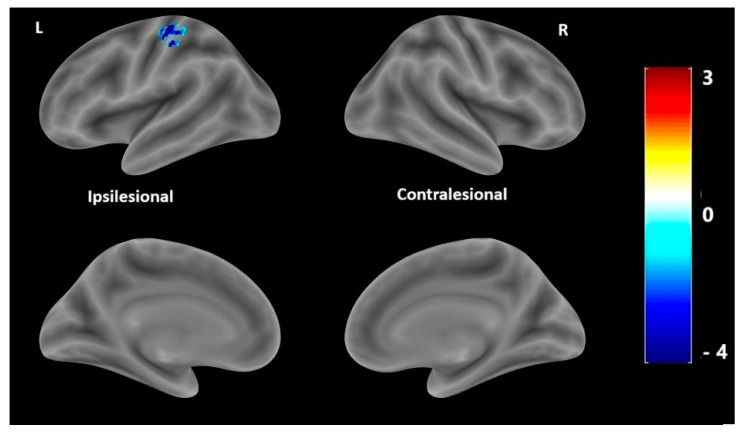
Statistical parametrical maps of activation during motor paradigm with the parietic hand, correlated with the lesion size. Cold colors indicate a negative correlation, specifically reflecting stronger activation in the left (ipsilesional) primary-sensory-motor cortex in individuals with smaller lesion sizes compared to those with larger lesions. There are no regions with a positive correlation of cortical activation with the lesion size (warm colors). Statistical parametric maps of activation are overlaid into a standard MNI template. The color bar represents Pearson’s Correlation coefficient. L: left (ipsilesional) hemisphere; R: right (contralesional hemisphere). BSPMview toolbox (http://www.bobspunt.com/software/bspmview, accessed on 21 September 2021) was used for results presentation, with threshold set at *p* < 0.05 (FWE-corrected).

The correlation analysis of brain lesion size revealed a negative correlation between lesion size and activation in the left ipsilesional primary-motor-sensory cortex (pre- and postcentral gyrus) and the right contralesional cerebellum. Specifically, patients with smaller lesions exhibited higher activation in the ipsilesional (contralateral to the hand) primary motor and somatosensory cortex, as well as the contralesional (ipsilateral to the hand) cerebellum (Table 4 and Figure 3).

**Figure 4:**
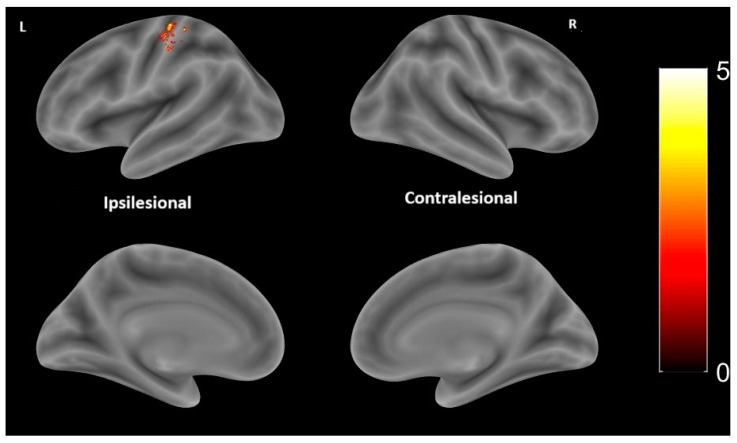
Statistical parametrical maps of activation in contrast subcortical > cortical lesions during motor paradigm with the paretic hand (warm colors), specifically reflecting stronger activation in the left (ipsilesional) primary-sensory-motor cortex in individuals with subcortical lesions compared to those with cortical lesions. No suprathreshold activation is found in the opposite contrast (cortical > subcortical lesions) (cold colors). Statistical parametric maps of activation are overlaid into a standard MNI template. The color bar represents the T-value from the test subcortical > cortical lesions. L: left (ipsilesional) hemisphere; R: right (contralesional hemisphere). BSPMview toolbox (http://www.bobspunt.com/software/bspmview, accessed on 21 September 2021) was used for results presentation, with a threshold set at *p* < 0.05 (FWE-corrected).

When comparing patients with cortical lesions to those with subcortical lesions, significantly higher activation was observed in the left (ipsilateral) postcentral gyrus in the subcortical lesions group. Results are shown in Table 5 and Figure 4.

A correlation analysis of brain activation during a motor task involving the paretic hand and the timing of lesion onset (when the brain injury occurred) was performed using SPM12 software to examine whether timing affected cortical organization. However, the results of this analysis showed that the timing of lesion onset did not significantly influence brain activation during the task (there was no suprathreshold activation).

## 4. Discussion

### 4.1. Sample Characteristics

Our study provides a large sample of patients with UCP, evaluated with both structural and functional MRI. We believe the sample size is one of the key strengths of this study, enhancing the statistical power of the analysis. Another contribution is the diversity of structural lesions, which allowed us to compare not only cortical and subcortical lesions, but also subgroups classified according to one of the latest MRI classifications. Additionally, our patient sample includes lesions acquired at four distinct time points during human brain ontogenesis: <24 weeks GA, 24–34 weeks GA, >34 weeks GA, and >1 month postnatally. These characteristics of the studied sample may also account for certain differences compared to previous similar studies [24].

In the group of patients we studied, right-side involvement (68% of patients enrolled in the study) predominated, similar to other studies [25]. The prevalence of right-sided UCP can be discussed as a result of the higher incidence of AIS in the left-middle cerebral artery [26]. So far, no significant differences have been reported between right-sided and left-sided hemiparesis, both in terms of motor and intellectual function, and neuroradiological findings [27,28,29], which allowed for the combined analysis of the two groups (after flipping the images with a lesion in the right hemisphere).

Among the participants in the study, there was a higher prevalence of MACS I and a lower prevalence of MACS II and III, in contrast to the study by Elliason et al. for scale validation [30]. Similar to the results of Carnahan et al., who reported 54% MACS I in hemiparesis patients [31]. We believe that these differences may result from disparities within the patient group and sample size. Also, the current study included only patients with mild to moderate disability due to the specific inclusion criteria.

Normal intelligence was observed in 64% of the patients studied. Due to the specific criteria of the study, only patients with mild intellectual disability (IQ ≥50) and acquired reading skills (mental age over 7 years) were included in the group. Our findings align with those of Rodopska’s local study, which reported normal intelligence in 54.5% of patients and mild intellectual disability in 33.6% [32], as well as Reid’s study in which normal intelligence was detected in 74.5% of patients with hemiplegia [33].

### 4.2. Lesion Characteristics

In our patient group, there was an equal number of patients with cortical and subcortical lesions—22 patients each. A cortical lesion was defined in all patients with MCA infarction (C3), all disorders of cortical formation (A1), and three-quarters of patients from the MRICS category D. The subcortical lesions group included all B-categories in MRICS, as well as two patients with lenticulostriate arteries infarcts (C1), and one postencephalitic patient with a residual basal ganglia lesion (D). When classified according to MRICS, vascular lesions (AIS, PVHI, and a combination of PVL + PVHI) constituted the majority of the lesions, accounting for a total of 32 patients (72.7%). These results confirm previous findings identifying perinatal strokes as the leading cause of congenital hemiplegia [34].

Cortical lesions were found to be larger in volume, while subcortical lesions exhibited significantly smaller volumes. The analysis revealed that this relationship was primarily driven by the AIS group, which showed the largest volume, and by PVL, which demonstrated the smallest volume. This finding is consistent with observations made by other authors [2,6]. Our study also found that postnatal lesions were second in size to AIS. Among the periventricular lesions, the largest volume was observed in the combined PVL + PVHI group, followed by PVHI, while the smallest volume was found in PVL alone. Despite these differences, the analysis for dependence between lesion type and functional deficits did not establish a significant relationship between the MACS level and the type of lesion defined as cortical or subcortical or by MRICS. Similar are the results of Holmstrom et al. and Holmefur et al., who assumed that motor function did not depend on and could not be explained by the type of lesion alone [9,35]. Himmelman et al. also found no significant difference in motor function in patients with UCP with different types of MRICS lesions [36]. In contrast, Feys et al. revealed a better motor function for patients with periventricular lesions and found no difference between patients with perinatal and later acquired cortico-subcortical infarctions [7]. Like them, Mackey et al. and Staudt et al. in independent studies found better motor function in patients with periventricular lesions and malformations compared to those with cortico-subcortical infarctions but attributed it to the time of occurrence of the lesion [2,37]. Cioni et al. found better motor performance in patients with both PVL and postnatally acquired lesions compared to those with malformations or perinatal AIS [3]. Our group of patients with brain malformations, e.g., time of lesion before 24 weeks GA, is small (only three individuals) and this could influence the result of no correlation between lesion timing and motor deficit.

We found that patients with cortical lesions exhibited a significantly higher incidence of mirror movements, a phenomenon also observed by other authors, with a variable frequency of up to 60% [38,39]. One possible explanation for this occurrence is bilateral activation in M1, leading to simultaneous movement of both hands [40,41]. As an anatomical substrate for pathological synkinesis, the impaired integrity of the corpus callosum [41,42,43] or the persistence of ipsilateral of the affected limb corticospinal fibers have been discussed [12,44,45,46]. In a previous study that investigated individual data from patients with UCP, we also found significantly higher contralesional M1 activation in patients with cortical lesions, which could serve as the substrate for their MMs [47].

The correlation analysis between the volume of the lesion and the clinical indicators found a significant positive correlation with motor functional deficit of the upper limb, as defined by the MACS level. This means that as the size of the lesion increased, the severity of the motor deficit in the affected upper limb also became more pronounced. Specifically, individuals with larger brain lesions were more likely to be classified at higher MACS levels, indicating greater limitations in their ability to handle objects and perform daily activities with the affected hand. This finding aligns with the existing literature [2,8,9,48], regardless of the different methods used to determine the size of the lesion and different assessment scales for motor deficiency—GMFCS, MACS, or others. Larger lesions are likely to disrupt more critical motor control areas in the brain, leading to greater functional impairment in hand usage. This hypothesis is further supported by the fMRI results of our study. From a clinical perspective, our findings suggest that patients with larger lesions are at a disadvantage in terms of achieving optimal motor function, so they need earlier and more intensive rehabilitation interventions. Larger lesions correlated also with the presence of MM, which suggests that they may disrupt neural mechanisms involved in motor control and inhibition, causing unintended movement in the non-affected hand during intentional movement of the affected hand. This finding and hypothesis are further supported by other studies [17,49].

The timing of the lesion is a widely discussed topic in the literature due to the hypothesis of different reorganization potentials of the brain at different stages of its development [2,50,51,52,53,54,55]. In most cases, a presumed time of occurrence is determined based on patterns of brain substance damage. The most widespread classification for the time of occurrence of lesions is as follows: white matter lesions (PVL or PVHI) occur during the early third trimester; and gray matter lesions in the late third trimester or perinatally [2,5,56]. The conditionality of this classification is emphasized due to existing data on the occurrence of lesions in the gray matter even in an earlier period (e.g., AIS in premature newborns, fetal ischemic infarction) [57,58]. To determine the time of lesion onset in our study, we combined morphological characteristics (lesion type by MRICS) with data on specific events that may have contributed to lesion development. For instance, two patients with AIS who were premature (<34 weeks gestation) were classified as having lesions acquired in the early third trimester, while two other patients with AIS had postnatally acquired lesions (ischemic stroke > 30 days of age). All patients with B-type lesions (PVL, PVHI, or a combination) were classified as having lesions acquired in the early third trimester, due to the unique blood supply characteristics in the periventricular regions, which suggest that such lesions typically occur during this period. In our study group, the timing of lesion onset did not show any association with subsequent clinical deficits, including the severity of motor involvement (MACS level), MM, or hand-clapping speed. In contrast, Staudt et al. found a significant association between lesion onset and motor function of the affected limb, with the most favorable motor function observed in patients with malformations (lesions occurring in the early prenatal period), followed by PVL, acquired between 24 and 34 weeks, and the poorest outcomes seen in patients with AIS (acquired after 34 weeks). In their study, the authors suggested that these differences could be attributed to variations in lesion size [2]. Our study could support the latter fact, because AIS had a larger size than PVL, so the size could be the determining factor for motor capacity after an early unilateral brain lesion. Another factor that might influence the results could be the small number of patients with malformations of cortical development (lesion timing before 24 weeks GA) included in our study. Although there was no significant difference, we did observe a predominance of MACS I in the group with lesions acquired earlier prenatally and a predominance of MACS III in those with later prenatal lesions. However, in patients with postnatally acquired lesions, good motor function (MACS I) again predominated, which raises questions about the role of lesion timing alone in determining a patient’s rehabilitation potential. Feys et al. reported similar findings with no significant difference in motor performance between patients with congenital and acquired ischemic strokes [7]. Cioni et al. even found better motor function in patients with postnatal lesions compared to those with malformations or perinatal strokes [3]. A possible explanation could be the differences in the volume of postnatal lesions compared to perinatal lesions, as observed in our study.

The differences observed, both in previous studies and in the present one, highlight the need for larger, even multicenter studies, on the effect of lesion onset timing on motor function regarding the other possible factors that can influence motor potential.

Discovering all factors that correlate with the motor function of patients with UCP could lead to the optimization of rehabilitation techniques applied to children with UCP.

### 4.3. Functional Reorganization in the Motor Cortex of UCP Patients

The relationship between the morphological characteristics of the lesion and the functional reorganization in the motor cortex is even more complex. Our study found a significant negative correlation between the size of the lesion and the volume of activation in the ipsilesional motor cortex. More specifically, larger lesions activated the ipsilesional (contralateral to the examined limb) M1 to a lesser extent. Conversely, small lesions showed significant activation in the primary motor cortex, and in the cerebellum contralaterally (ipsilesionally)—similar to activation in healthy individuals [59]. Similar results were reported in animal models [60,61,62] which also showed higher activation in the contralesional motor cortex, a finding not observed in our patient group. Staudt et al. also commented on the difference in motor activation depending on the size of the lesion, noting a relationship between the degree of ipsilesional reorganization and lesion size [17]. However, his study focused solely on patients with PVL, while our current study demonstrated that this relationship holds across a broader range of lesion types. The observed results indicate that larger lesions may disrupt critical motor control areas in the brain, potentially accounting for the impaired hand function observed in patients with more extensive brain damage.

We also explored the dependencies of motor activation in relation to other morphological characteristics of the lesion. Group-level comparisons revealed significantly higher activation in the ipsilesional M1S1 in patients with subcortical lesions compared to those with cortical lesions. No differences in activation were observed in regions other than M1S1. These results are in line with the findings by Mackey et al., who also reported dominant ipsilesional reorganization in patients with PVL, with variable activation observed in patients with malformations and AIS [37]. Unlike Staudt et al., Vandermeeren et al., and Mackey et al., we did not find significant contralesional activation in patients with cortical lesions [2,23,37]. A possible explanation for this finding could be the variability of motor-system reorganization in patients with cortical lesions. The individual involvement of different cortical regions may prevent sufficiently high, statistically significant activation in a single structure at the group level [47].

The relationship between the type of reorganization in the motor cortex and the timing of the lesion has been widely discussed in the literature. However, our group-level analysis did not find a significant difference in brain activation based on the time of lesion onset, whether early antenatal, early prenatal, late prenatal/intranatal, or postnatally acquired. According to the existing literature, contralesional (ipsilateral to the hand) corticospinal projections may only be preserved in cases of earlier insults [14]. According to Staudt’s studies [2], the possibility of contralesional reorganization decreases even in patients with lesions acquired at the end of the third trimester. This is further supported by Batschelett et al., which found interhemispheric motor reorganization in 60.7% of patients with acquired injuries and only 15.8% of patients with developmental disorders [20]. However, none of these studies focus exclusively on fMRI and include data from other methods such as EMG or TMS. Additionally, no study has compared brain activation across four distinct periods of early development during which the lesion was acquired. The possibility of bilateral corticospinal projections existing even in the postnatal period (as demonstrated in Eyre’s studies [14]) defines the potential for the development of different reorganization patterns, regardless of the timing of lesion onset. This is further supported by the current study. Considering our results, we believe that the brain’s potential for reorganization is sufficient to achieve good functional outcomes even in the case of early postnatally acquired lesions and the leading determined factor is brain lesion size. However, a limitation of our study is the small number of patients in the ’early antenatal period’ category (only three) and those in the ’postnatally acquired’ category (six).

## 5. Limitations and Future Directions

The sample characteristics of our study constitute a notable strength, particularly regarding the sample size and diversity. A substantial cohort of UCP patients were assessed using both structural and functional MRI, thereby providing a comprehensive dataset for analysis. Given the multifaceted nature of the condition, this large dataset includes a heterogeneous group of patients varying in age, gender, cultural and socioeconomic background, motor performance, concomitant conditions, and lesion characteristics. While this diversity enhances the generalizability of the findings, it also introduces heterogeneity that may reduce the statistical power of the results.

Given the nature of fMRI and its requirement for patient cooperation, we restricted our inclusion to individuals with mild to moderate hand function impairments (MACS I-III) and mild to moderate intellectual disabilities that could present some limitation of the study.

While some studies have reported better motor outcomes in patients with earlier lesions, our study indicates that the brain may retain sufficient reorganization potential even with postnatally acquired lesions. However, the small number of patients in certain categories (e.g., early antenatal and postnatally acquired lesions) warrants caution, and larger, multicenter studies might be needed to explore the effects of lesion timing in more depth.

In future studies, a larger sample size and an enhancement of the homogeneity of patient groups are needed to strengthen the statistical power of the results, facilitating their inclusion in clinical applications to optimize individual motor potential.

Future study should explore the impact of different rehabilitation techniques, testing the hypothesis that early and more intensive physical therapy can optimize rehabilitation outcomes and maximize the potential for functional recovery.

## 6. Conclusions

This study reinforces prior research indicating that perinatal-ischemic strokes are the primary cause of congenital hemiplegia, with a significant number of patients exhibiting cortical lesions. Interestingly, despite the diversity of the lesions, no clear correlation was found between lesion type (cortical vs. subcortical, or by MRICS) and motor function severity as measured by MACS levels. Similarly, no definitive relationship was observed between the timing of the lesion and motor function outcomes. The most significant predictor of motor performance in our study was lesion size, with larger lesions being associated with poorer motor function. This finding suggests a promising direction for future research and clinical strategies focused on customizing rehabilitation approaches such as early and intensive interventions for patients with larger lesions, regardless of whether they occurred prenatally or postnatally before 18 months of age. Optimizing rehabilitation strategies could maximize functional outcomes, irrespective of lesion timing. Ultimately, the variability in individual brain reorganization and the influence of lesion characteristics on motor performance underscores the necessity of adopting more personalized approaches for understanding and managing UCP.

Motor functional reorganization in UCP is closely linked to lesion characteristics, with smaller, subcortical lesions favoring typical organization in the contralateral motor cortex, while larger, cortical lesions do not demonstrate significant group activation, likely due to individual differences in neural reorganization. The altered contralateral motor control organization in patients with larger lesions may contribute to the more severe motor impairments observed in these individuals. The timing of the lesion does not significantly affect cortical reorganization, determining the brain’s ability to reorganize motor pathways even if the lesion is acquired early postnatally.

Further studies with larger and more homogeneous groups could help clarify the interactions between the variables and enhance therapeutic strategies.

## Figures and Tables

**Table 1:** Individual clinical and imaging characteristics of the participants in the study.

Patient	Affected Side	MRICS	Lesion Type	Lesion Volume (mL)	Time of Occurrence	MACS	IQ
1	L	C3	Cortical	45.978	Late prenatal	2	90
2	R	B2	Subcortical	42.039	Early prenatal	1	98
3	R	C3	Cortical	264.175	Late prenatal	3	78
4	R	C3	Cortical	254.09	Late prenatal	3	98
5	R	C3	Cortical	267.736	Late prenatal	3	60
6	R	C3	Cortical	106.886	Late prenatal	2	80
7	R	D	Cortical	52.022	Postnatal	1	78
8	R	B1	Subcortical	9.48	Early prenatal	1	78
9	R	C3	Cortical	162.469	Late prenatal	1	89
10	R	B2	Subcortical	31.847	Early prenatal	3	50
11	R	C3	Cortical	91.959	Early prenatal	2	78
12	R	B1	Subcortical	4.546	Early prenatal	1	58
13	L	B2	Subcortical	27.456	Early prenatal	3	65
14	R	A1	Cortical	84.088	Very early prenatal	2	88
15	R	A1	Cortical	92.347	Very early prenatal	1	98
16	L	C3	Cortical	170.318	Late prenatal	3	88
17	R	C3	Cortical	81.813	Late prenatal	1	95
18	R	B3	Subcortical	4.809	Early prenatal	1	87
19	L	D	Subcortical	67.753	Postnatal	1	58
20	R	B3	Subcortical	10.314	Early prenatal	1	67
21	L	B1	Subcortical	4.843	Early prenatal	1	73
22	R	C1	Subcortical	6.17	Postnatal	3	98
23	R	B2	Subcortical	16.16	Early prenatal	1	81
24	L	C3	Cortical	259.777	Late prenatal	3	75
25	R	B1	Subcortical	8.363	Early prenatal	3	60
26	L	B2	Subcortical	20.523	Early prenatal	3	92
27	R	C3	Cortical	192.024	Early prenatal	3	68
28	L	C3	Cortical	258.698	Late prenatal	3	62
29	R	B2	Subcortical	29.815	Early prenatal	2	65
30	L	B3	Subcortical	49.68	Early prenatal	1	103
31	L	D	Cortical	199.822	Postnatal	3	58
32	R	C1	Subcortical	29.872	Late prenatal	1	88
33	R	B2	Subcortical	15.282	Early prenatal	1	103
34	R	B2	Subcortical	10.051	Early prenatal	1	88
35	L	B3	Subcortical	47.903	Early prenatal	3	53
36	R	C3	Cortical	371.102	Late prenatal	3	60
37	R	C3	Cortical	179.533	Late prenatal	1	98
38	R	B2	Subcortical	8.424	Early prenatal	2	103
39	L	A1	Cortical	75.337	Very early prenatal	3	68
40	L	C3	Cortical	31.85	Late prenatal	1	98
41	R	D	Cortical	126.4	Postnatal	1	78
42	L	C3	Cortical	422.087	Late prenatal	3	60
43	R	B1	Subcortical	28.036	Early prenatal	1	62
44	R	C1	Subcortical	5.957	Postnatal	1	102

MRICS: Magnetic Resonance Imaging Classification System; MACS: Manual Ability Classification System; IQ: Intelligence Quotient; L: Left; R: Right; A1: disorders of cortical formation; B1: periventricular leukomalacia; B2: sequelae of intraventricular hemorrhage or periventricular hemorrhagic infarction; B3: combination of periventricular leukomalacia and periventricular hemorrhagic infarction sequelae; C1: basal ganglia/thalamus infarctions; C3 lesions: arterial ischemic infarction in the middle cerebral artery; D: miscellaneous.

**Table 2:** Correlation coefficient for lesion size and motor performance measurements. MACS: manual Ability Classification System; MM: mirror movements; **: significant correlation; NS: non-significant correlation.

Lesion Size	Spearman’s Rho	*p*
MACS	0.440 **	<0.05
MM	0.534 **	<0.05
Hand-clapping speed	0.172	NS

**Table 3:** fMRI results of brain activation during the motor task with the paretic hand in patients with UCP. There is significant activation in the ipsilesional motor cortex and the cerebellum bilaterally. T-stat: T-value; N: number; MNI: Montreal Neurological Institute.

Activated Clusters			MNI Coordinates		
Region	N Activated Voxels	T-Stat	x	y	z
Left precentral gyrus	426	7.300	−28	−22	66
Left cerebellum (Lobul 1)	313	8.852	−6	−84	−12
Left cerebellum (Lobul VI)	144	7.912	−26	−66	−22
Right cerebellum (Lobul VI)	188	7.003	16	−86	−14
Cerebellar vermis	251	6.768	2	−64	−18

**Table 4:** fMRI results, showing correlation analysis between activation during the motor paradigm with the paretic hand in UCP patients and lesion size. R: Pearson’s correlation coefficient; N: number; MNI: Montreal Neurological Institute.

**Clusters with Significant Activation in Correlation with Lesion Size and fMRI Activation During the Motor Paradigm (Positive)**			**MNI Coordinates**		
None			-		
**Clusters Showing Significant Activation in Correlation with Lesion Size and fMRI Activation During the Motor Paradigm** **(Negative)**			**MNI Coordinates**		
Region	N of activated voxels	R	x	y	z
Left pre- and postcentral gyrus	383	−4.037	−34	−32	54
Right cerebellum	18	−4.152	6	−50	−40

**Table 5:** fMRI results, showing significant clusters of activation in the comparison between patients with subcortical and cortical lesions during the motor paradigm with the paretic (uniformly right) hand. N: number; MNI: Montreal Neurological Institute.

**Clusters with Significant Activation** **(Contrast Subcortical > Cortical Lesions)**			**MNI Coordinates**		
**Region**	**N of Activated Voxels**	**T-Value**	**x**	**y**	**z**
Left precentral gyrus	156	5.431	−34	−30	54
Left postcentral gyrus	152	4.473	−54	−22	48
**Clusters with significant activation** **(Contrast cortical > subcortical lesions)**			**MNI** **coordinates**		
None			-	-	-

## Data Availability

The datasets presented in this article are not readily available due to technical limitations (large dataset). The raw data supporting the conclusions of this article will be made available by the authors on request.

## Abbreviations

The following abbreviations are used in this manuscript:

UCP	Unilateral cerebral palsy
fMRI	Functional Magnetic Resonance Imaging
MACS	Manual Ability Classification System
AIS	Arterial Ischemic Stroke
IQ	Intelligence Quotient
M1	Primary Motor Cortex
S1	Primary Sensory Cortex
ICD-10	International Classification of Diseases—10th edition
GMFCS	Gross Motor Function Classification System
3D	Three-Dimensional
2D	Two-Dimensional
T1	Longitudinal Relaxation Time Imaging
FSPGR	Fast Spoiled Gradient-Echo
MRICS	Magnetic Resonance Imaging Classification System
DICOM	Digital Imaging and Communications in Medicine
SPM	Statistical Parametric Mapping
MNI	Montreal Neurological Institute
BOLD	Blood Oxygen Level Dependent
LST	Lesion Segmentation Tool
EPI	Echo-planar imaging
TR	Repetition Time
TE	Echo Time
FWE	Family-Wise Error
PVL	Periventricular Leukomalacia
IVH	Intraventricular Hemorrhage
PVHI	Periventricular Hemorrhagic Infarction
MM	Mirror Movements
CSF	Cerebro-Spinal Fluid
GA	Gestational Age

## Appendix A

**Table A1:** Associations between clinical motor performance and lesion type, categorized as cortical and subcortical.

Motor Function Determinants			Lesion Type			X^2^	df	*p*
			Subcortical	Cortical	All			
MACS	1	N	14	7	21	4.471	1	>0.05
		%	63.6%	31.8%	47.7%			
	2	N	2	4	6			
		%	9.1%	18.2%	13.6%			
	3	N	6	11	17			
		%	27.3%	50%	38.6%			
MM *	Yes	N	4	11	15	4.956	1	<0.05
		%	18.2%	50%	31.8%			
	No	N	18	11	29			
		%	82.8%	50%	68.2%			
Hand-clapping speed	Normal	N	4	1	5	2.031	1	>0.05
		%	18.2%	4.5%	11.3%			
	Delayed	N	18	21	39			
		%	82.8%	95.5%	88.7%			

MACS: Manual Ability Classification System; MM: Mirror Movements; N: Number; *: significant association.

**Table A2:** Associations between motor performance determinants and lesion type, categorized according to MRICS.

Motor Function Determinants	N (%)	Lesion Type (MRICS)							X^2^	df	*p*
		A1 (DCF)	B1 (PVL)	B2 (PVHI)	B3 (PVL + PVHI)	C1 (BG)	C3 (AIS)	D (Misc)			
MACS	1	1 (33.3%)	4 (80%)	4 (44.4%)	3 (25%)	2 (66.7%)	4 (25%)	3 (75%)	10.503	12	NS
	2	1 (33.3%)	0 (0%)	2 (22.2%)	0 (0%)	0 (0%)	3 (18.7%)	0 (0%)			
	3	1 (33.3%)	1 (20%)	3 (33.3%)	1 (25%)	1 (33.3%)	9 (56.3%)	1 (25%)			
MM	No	1 (33.3%)	2 (40%)	3 (33.3%)	2 (50%)	2 (66.7%)	2 (12.5%)	3 (75%)	8.247	6	NS
	Yes	2 (66.7%)	3 (60%)	6 (66.7%)	2 (50%)	1 (33.3%)	14 (87.5%)	1 (25%)			
Hand-clapping speed	Normal	0 (0%)	1 (20%)	1 (11.1%)	2 (50%)	0 (0%)	0 (0%)	1 (25%)	9.858	6	NS
	Delayed	3 (100%)	4 (80%)	8 (88.9%)	2 (50%)	3 (100%)	16 (100%)	3 (75%)			

MACS: Manual Ability Classification System; MM: mirror movements; N: number; A1: disorders of cortical formation (DCF; B1: periventricular leukomalacia (PVL); B2: sequelae of intraventricular hemorrhage (IVH) or periventricular hemorrhagic infarction (PVHI); B3: combination of periventricular leukomalacia and periventricular hemorrhagic infarction sequelae; C1: basal ganglia (BG)/thalamus infarctions; C3 lesions: arterial ischemic stroke (AIS) in the middle cerebral artery; D: miscellaneous (Misc); NS: non-significant.
